# Early-onset breast cancer in two Brazilian tertiary cancer centers: a real-world study of years of life lost and clinical outcomes

**DOI:** 10.3389/fonc.2026.1837330

**Published:** 2026-10-27

**Authors:** Diego Lopes Paim Miranda, Douglas Dias e Silva, Beatriz Viesser Miyamura, Mariana de Moura de Souza, Maria Fernanda Teixeira, Uelson Donizeti Rocioli Junior, Fernando Moura, Pedro Luiz Serrano Uson Junior, Patricia Taranto, Janaina Pontes Batista, Max Senna Mano, Rafael Aliosha Kaliks Guendelmann, Heloisa Veasey Rodrigues, Juliana Rodrigues Beal

**Affiliations:** 1 Department of Oncology, Hospital Israelita Albert Einstein, São Paulo, Brazil; 2 Center for Precision Medicine, Hospital Israelita Albert Einstein, São Paulo, Brazil

**Keywords:** Brazil, breast cancer epidemiology, cancer burden, DALY, early-onset breast cancer, real-world data, years of life lost, young women

## Abstract

**Introduction:**

Early-onset breast cancer (EOBC) represents a growing global concern, particularly in low- and middle-income countries, where epidemiological data remain scarce. We evaluated the clinical characteristics, temporal trends, and disease burden of EOBC in two tertiary cancer centers in São Paulo, Brazil, with a focus on Years of Life Lost (YLL) and Disability-Adjusted Life Years (DALY).

**Methods:**

We conducted a retrospective study of patients diagnosed with malignant breast neoplasms between 2018 and 2023 at two tertiary cancer centers in São Paulo, Brazil. EOBC was defined as diagnosis in adult patients (aged ≥18 years) younger than 50 years. Demographics, clinical stage, and germline testing rates were compared between younger (<50) and older (≥50) patients. YLLs were calculated using individualized life expectancy estimates, and DALYs were computed as YLL + Years Lived with Disability (YLD). Group comparisons used chi-squared and Wilcoxon rank-sum tests, and multivariable logistic regression was used to evaluate factors associated with YLL >0.

**Results:**

Among 3,102 patients, 43.5% were aged <50 years. EOBC patients had lower median BMI (24.6 vs. 26.1 kg/m²; p<0.001) and higher germline testing rates (21.3% vs. 11%; p<0.001). Between 2018 and 2023, the proportion of breast cancer diagnoses among patients aged <50 years varied over time, but no statistically significant temporal trend was identified (p=0.30). The distribution of non-metastatic (stages I–III) versus metastatic (stage IV) disease was similar between age groups (p=0.14). Among patients who died during follow-up, median YLL was twice as high among patients aged <50 years compared with those aged ≥50 years (42 vs. 21 years). DALY rates per 1,000 patients were 33% higher among younger patients (3,340.8 vs. 2,509.3), primarily driven by a higher rate of YLL. In multivariable analysis, treatment at the participating public institution remained associated with higher odds of YLL >0 (adjusted OR 2.15; 95% CI 1.18–3.94; p=0.013).

**Conclusion:**

Within two tertiary cancer centers in São Paulo, Brazil, the proportion of EOBC diagnoses varied over the study period, without a statistically significant temporal trend. Patients aged <50 years had a greater aggregate burden of premature mortality, reflected by higher YLL and DALY rates, primarily due to the greater number of potential life years lost when death occurs at younger ages, despite a similar distribution of non-metastatic versus metastatic disease at diagnosis. These findings support the need for further research and may help inform age-specific strategies for the care of younger patients with breast cancer.

## Introduction

Breast cancer remains the most commonly diagnosed cancer and the leading cause of cancer-related death among women worldwide, accounting for approximately 685,000 deaths each year (1). In Brazil, breast cancer has overtaken cervical cancer as the leading cause of oncologic mortality among women (2). Although the majority of cases occur in women aged 50 years and older, a growing body of evidence suggests an increasing incidence among younger adults, particularly those under 50 (3, 4). This trend is concerning due to the potential for more aggressive tumor biology, delayed diagnoses, and the distinct clinical and psychosocial challenges faced by younger patients (5).

Despite growing concern, data on the epidemiology and clinical impact of early-onset breast cancer (EOBC) in low- and middle-income countries remain scarce (6). In Brazil and other Latin American nations, cancer registries often lack the detailed data necessary to analyze age-specific trends and outcomes effectively (7). Consequently, it remains unclear whether the observed rise in diagnoses among younger women reflects a true epidemiological trend, improved detection, or changes in exposure to risk factors. Additionally, there is limited knowledge on how EOBC affects long-term outcomes such as survival, disease progression, and Years of Life Lost (YLLs) in the Brazilian context.

To address these knowledge gaps, we conducted a retrospective real-world analysis using data from two tertiary cancer centers in Brazil. We evaluated the distribution of breast cancer cases diagnosed between 2018 and 2023, comparing clinical presentation, demographics, and outcomes between patients aged <50 and those aged ≥50 years. We also examined temporal trends in diagnosis and assessed the burden of premature mortality by calculating YLLs.

The primary aim of this study was to characterize the clinical and demographic profile of EOBC and compare it with that of older patients. Secondary objectives included evaluating temporal trends in the proportion of EOBC diagnoses within the participating centers, the extent of germline testing, and quantifying the impact of breast cancer on premature mortality among younger patients. As this was an institutional cohort, the study was not designed to estimate population-based incidence.

## Materials and methods

We conducted a retrospective observational study using real-world data collected from two tertiary cancer centers in the state of São Paulo, Brazil: Hospital Israelita Albert Einstein (a private hospital) and Hospital Municipal Vila Santa Catarina (a public hospital), covering the period from 2018 to 2023. The study included patients with malignant breast neoplasms (ICD-10 codes C50.0-C50.9). Patients with carcinoma *in situ* (ICD-10 code D05) were not included. Only patients with an initial diagnosis of breast cancer were included. Patients with recurrent disease were excluded. Individuals referred from other institutions were eligible, provided they were receiving initial oncologic management at one of the participating centers. Clinical stage corresponded to the stage at the initial breast cancer diagnosis, as documented at the time of the patient’s first evaluation at the participating institution. EOBC was defined as breast cancer diagnosed in adult patients (aged ≥18 years) younger than 50 years (<50 years), consistent with a recently proposed definition for sporadic early-onset cancers in adults (8).

Data extraction was performed through an automated process using the hospitals’ electronic medical record (EMR) systems. Because both institutions use the same electronic medical record platform, duplicate patients were identified using unique patient identifiers and consolidated before analysis. Clinical and demographic variables extracted included age at diagnosis, sex, body mass index (BMI), smoking status, alcohol use, germline testing status, clinical stage, and survival information. Smoking status and alcohol use were classified according to the information documented in the electronic medical records; no standardized quantitative thresholds for smoking or alcohol consumption were available.

Vital status was ascertained using information recorded in the institutional electronic medical records and cross-checked against the Brazilian Federal Revenue Service (Receita Federal) CPF registry using patients’ CPF numbers and dates of birth. Patients with a recorded death in either source were classified as deceased. Patients without a recorded death were considered alive at their last available clinical follow-up and were censored on that date. Median follow-up was calculated from the date of breast cancer diagnosis to the date of death or last available follow-up, as applicable.

The study was approved by the Research Ethics Committee of Hospital Israelita Albert Einstein under CAAE 77778823.9.0000.0071. The requirement for written informed consent was waived because data were obtained through retrospective extraction of anonymized electronic medical records.

For the calculation of YLLs, individual projected life expectancy estimates were obtained from the 2022 Complete Life Tables published by the Brazilian Institute of Geography and Statistics (IBGE) (9). For each patient, projected life expectancy was assigned according to year of birth, sex, and place of residence.

In addition, Disability-Adjusted Life Years (DALYs) were calculated as the sum of YLLs and Years Lived with Disability (YLDs). For patients who died during follow-up, YLL was calculated as the difference between projected life expectancy and age at death, with negative values truncated at zero. Patients who remained alive at the end of follow-up were assigned a YLL value of zero.

YLL totals, rates per 1,000 patients, and patient-level comparisons between age groups were calculated using the entire cohort, including survivors with YLL equal to zero. In contrast, the YLL medians and interquartile ranges reported for the age groups, as well as the distributions, were calculated exclusively among patients who died during follow-up.

YLDs were calculated by multiplying the duration of illness, defined as the interval between the date of diagnosis and the date of last clinical follow-up or death, by a disability weight assigned according to disease stage. Disability weights were based on the provisional Global Burden of Disease (GBD 2000) cancer disability weights, which assign disability weights of approximately 0.1 during diagnosis, primary therapy, and control phases and substantially higher weights for preterminal metastatic and terminal disease, which were simplified to 0.8 in the present study (10).

Because detailed longitudinal information on transitions between treatment phases, recurrence, remission, and disease progression was not consistently available in the electronic medical records, a simplified stage-based approach was adopted. Patients without documented metastatic disease (stages I-III) were assigned a disability weight of 0.1, whereas patients with metastatic disease (stage IV) were assigned a disability weight of 0.8. Since individual information for stages I-III was not available, disease stage was categorized as stages I-III versus stage IV.

Because YLD was calculated using the observed duration of follow-up, patients diagnosed earlier contributed longer periods of follow-up and therefore accumulated more YLD than those diagnosed more recently. This reflects the observed follow-up time available in this retrospective cohort and was not adjusted analytically.

Patients were categorized into two age groups: <50 years and ≥50 years. For YLL, YLD, and DALY, patient-level distributions were summarized using medians and interquartile ranges. The number and proportion of patients with values greater than zero, total values, and rates per 1,000 patients were also calculated for each age group.

Annual institutional breast cancer diagnosis rates were calculated by dividing the number of breast cancer diagnoses in each age group during a given calendar year by the total number of patients registered at the institution in that year and multiplying by 100,000. Temporal changes in the proportion of patients aged <50 years were evaluated using binomial logistic regression, with calendar year included as a continuous predictor.

Statistical analyses were performed using R (version 4.5.1). Categorical variables were summarized as frequencies and compared using Pearson’s chi-squared test. For contingency tables with small expected cell counts, p-values were estimated using Monte Carlo simulation with 20,000 replicates. Continuous variables were summarized as medians and interquartile ranges and compared using the Wilcoxon rank-sum test.

YLD was retained as a continuous outcome and was not dichotomized for regression analysis. YLL >0 was defined as a binary indicator of premature mortality. Logistic regression models were used to evaluate the association between the participating institution (public versus private hospital) and the probability of YLL >0. Unadjusted logistic regression models were performed separately within each age group (<50 and ≥50 years). A primary multivariable logistic regression model was then fitted with YLL >0 as the dependent variable and participating institution, age at admission, disease stage, BMI, ethnicity, smoking status, and alcohol use as covariates.

A sensitivity analysis additionally included germline and somatic testing status. Both multivariable models were conducted using complete-case analysis. Regression results were reported as odds ratios (ORs) or adjusted odds ratios (aORs), as appropriate, with 95% confidence intervals (CIs) and exact p-values. Statistical significance was set at p<0.05. YLL distributions were visualized using age-stratified histograms restricted to patients who died during follow-up.

## Results

A total of 3,102 patients were analyzed, including 1,752 (56.5%) aged ≥50 years and 1,350 (43.5%) aged <50 years. Women comprised 99.3% of the cohort, with a higher proportion of male patients among older individuals (1% vs. 0.3%; p=0.023). Baseline demographic and clinical characteristics stratified by age group are summarized in **Table 1**.

**Table 1:** Baseline demographic and clinical characteristics of patients diagnosed with breast cancer, stratified by age group (<50 vs ≥50 years).

Variables	Available N / missing, n (%)	< 50 N = 1,350¹	≥ 50 N = 1,752¹	p-value²
Sex	3,102 / 0 (0%)			**0.023**
Female		1,346 (99.7%)	1,735 (99.0%)	
Male		4 (0.3%)	17 (1.0%)	
Alcohol use	3,102 / 0 (0%)			0.12
No		572 (42.4%)	791 (45.1%)	
Yes		778 (57.6%)	961 (54.9%)	
Smoking	3,102 / 0 (0%)			0.6
No		536 (39.7%)	679 (38.8%)	
Yes		814 (60.3%)	1,073 (61.2%)	
Ethnicity	3,102 / 0 (0%)			0.069
Asian		17 (1.3%)	28 (1.6%)	
Black		24 (1.8%)	23 (1.3%)	
Mixed race		62 (4.6%)	85 (4.9%)	
Uninformed		975 (72.2%)	1,195 (68.2%)	
White		272 (20.1%)	421 (24.0%)	
Staging	3,102 / 0 (0%)			0.14
I, II, III		827 (61.3%)	1,027 (58.6%)	
IV		523 (38.7%)	725 (41.4%)	
BMI	1,940 / 1,162 (37%)			**<0.001**
Median (Q1–Q3)		24.6 (22.0–28.3)	26.1 (23.4–29.4)	
Min, Max		13.9, 53.1	15.4, 52.7	
Germline Test	3,102 / 0 (0%)			**<0.001**
No		1,063 (78.7%)	1,565 (89.3%)	
Yes		287 (21.3%)	187 (10.7%)	
YLD	3,102 / 0 (0%)			0.2
Median (Q1–Q3)		0.33 (0.04–1.39)	0.29 (0.02–1.29)	
Min, Max		0.00, 5.88	0.00, 5.98	
YLL	3,102 / 0 (0%)			0.066
Median (Q1–Q3)		0.0 (0.0–0.0)	0.0 (0.0–0.0)	
Min, Max		0.0, 53.8	0.0, 34.3	
DALY	3,102 / 0 (0%)			0.7
Median (Q1–Q3)		0.4 (0.1–1.9)	0.4 (0.0–2.2)	
Min, Max		0.0, 54.0	0.0, 34.6	

¹n (%) ²Pearson’s Chi-squared test; Wilcoxon rank sum test.

BMI, Body Mass Index;

Min, Minimum; Max, Maximum;

Q1, First quartile; Q2, Median; Q3, Third quartile;

YLD, Years Lived with Disability;

YLL, Years of Life Lost;

DALY, Disability-Adjusted Life Years.

Bold values indicate statistically significant results (p < 0.05).

Notably, younger patients had a lower median body mass index (BMI) (24.6 vs. 26.1 kg/m²; p<0.001) and were significantly more likely to undergo germline testing (21.3% vs. 10.7%; p<0.001).

Median follow-up was 28.25 months among patients aged <50 years and 27.10 months among those aged ≥50 years. During follow-up, 75 patients aged <50 years and 135 patients aged ≥50 years died.

Between 2018 and 2023, the proportion of breast cancer diagnoses among patients aged <50 years within the participating institutions varied over time, with fluctuations across individual years. No statistically significant temporal trend was identified (p=0.30). These temporal trends are illustrated in **Figure 1**.

**Figure 1:**
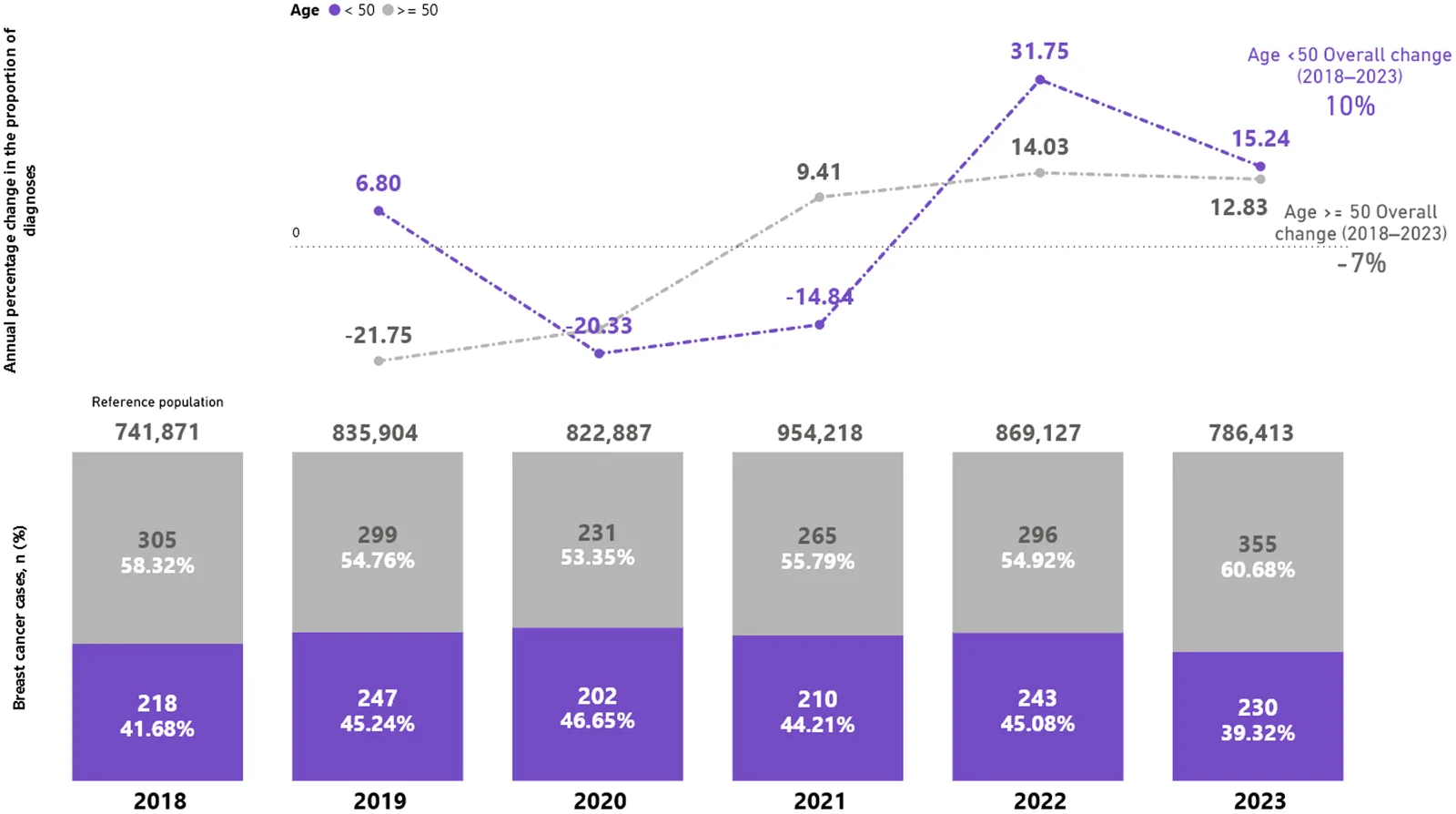
Temporal trends in the number and proportion of breast cancer diagnoses by age group (<50 vs ≥50 years) between 2018 and 2023. Bars show the number and proportion of diagnoses in each age group, while the upper panel shows the annual percentage change in the age-specific diagnosis rate. The reference population is shown above each bar. The cohort included 3,102 patients. Temporal trends in the proportion of patients aged <50 years were evaluated using binomial logistic regression with calendar year as a continuous predictor (p=0.3).

Clinical stage at diagnosis was similarly distributed in both groups, with localized or locally advanced disease present in 61.3% of younger and 58.6% of older patients (p=0.14).

When evaluating the burden of premature mortality using Years of Life Lost (YLL), among patients who died during follow-up, those aged <50 years had a median YLL of 42 years (interquartile range [IQR] 37.5–46.0), compared with 21 years (IQR 13.0–26.0) among patients aged ≥50 years. This difference reflects the greater number of potential life years lost when death occurs at younger ages. **Figure 2** illustrates the distribution of YLL among patients who died during follow-up. Patients aged <50 years had YLL values concentrated between 40 and 50 years, whereas those aged ≥50 years showed YLL values concentrated between 20 and 30 years.Aggregate measures of YLL, YLD, and DALY are presented in **Table 2**. The aggregate DALY rate per 1,000 patients was 33% higher in the younger group than in the older group (3,340.8 vs. 2,509.3), primarily driven by a 53% higher aggregate YLL rate (2,374.6 vs. 1,549.1 per 1,000 patients). In contrast, aggregate YLD rates per 1,000 patients were similar between groups (966.3 vs. 960.2). These aggregate rates were calculated using the full cohort, whereas patient-level distributions were assessed separately.

**Figure 2:**
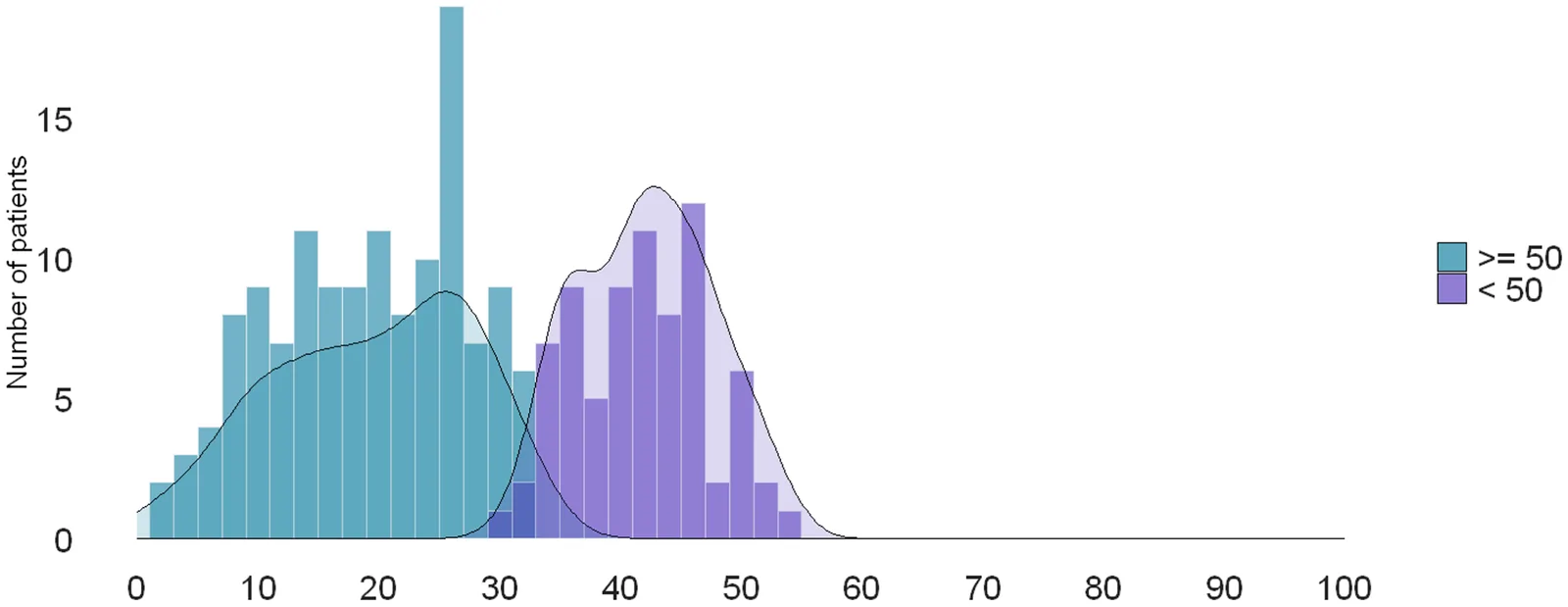
Distribution of Years of Life Lost (YLL) among patients who died during follow-up, stratified by age group (<50 vs ≥50 years). Histograms display the number of patients in each YLL interval. A total of 75 deaths occurred among patients aged <50 years and 135 among those aged ≥50 years.

**Table 2:** Patient contributions and aggregate burden of YLL, YLD, and DALY by age group.

Statistic	< 50	>= 50
YLL		
Nonzero values, n (%)	75 (5.6%)	134 (7.6%)
Median (IQR), years	0.0 (0.0–0.0)	0.0 (0.0–0.0)
Total, years	3205.7	2714.0
Rate per 1,000 patients	2374.6	1549.1
YLD		
Nonzero values, n (%)	1133 (83.9%)	1417 (80.9%)
Median (IQR), years	0.3 (0.0–1.4)	0.3 (0.0–1.3)
Total, years	1304.4	1682.3
Rate per 1,000 patients	966.3	960.2
DALY		
Nonzero values, n (%)	1151 (85.3%)	1453 (82.9%)
Median (IQR), years	0.4 (0.1–1.9)	0.4 (0.0–2.2)
Total, years	4510.1	4396.3
Rate per 1,000 patients	3340.8	2509.3

YLL, Years of Life Lost;.

YLD, Years Lived with Disability;

DALY, Disability-Adjusted Life Years;

IQR , Interquartile Range.

Nonzero values represent the number and proportion of patients with values greater than zero.

Rates per 1,000 patients were calculated as the total number of years divided by the number of patients in each age group and multiplied by 1,000.

Medians and interquartile ranges were calculated using patient-level distributions in the full cohort, including patients with zero values. For YLL, patients who remained alive at the end of follow-up were assigned a value of zero. In contrast, the median YLL values reported in the Results were calculated exclusively among patients who died during follow-up.

When evaluating premature mortality (YLL >0), logistic regression showed that care received at the participating public institution was associated with higher odds of YLL >0 among patients aged <50 years (OR 2.21; 95% CI 1.22–3.82; p=0.006) compared with the participating private institution. Similar patterns were observed among patients aged ≥50 years. In the primary multivariable complete-case analysis, which included 1,940 patients, treatment at the participating public institution remained associated with higher odds of YLL >0 after adjustment for age at admission, clinical stage, BMI, ethnicity, smoking status, and alcohol use (adjusted OR 2.15; 95% CI 1.18–3.94; p=0.013). Stage IV disease was also associated with higher odds of YLL >0 compared with stages I-III (adjusted OR 5.74; 95% CI 3.72–9.09; p<0.001). In a sensitivity analysis additionally adjusted for germline and somatic testing, the association between treatment at the participating public institution and YLL >0 remained statistically significant and similar in magnitude (adjusted OR 2.00; 95% CI 1.08–3.73; p=0.029). The results of the multivariable models are presented in **Table 3**. The observed proportions of YLL >0 were 4.9% vs. 10.2% (<50 years) and 6.9% vs. 12.7% (≥50 years) for the private and public participating institutions, respectively. Additional stratified analyses comparing outcomes between the public and private participating institutions are presented in **Table 4**. Detailed demographic and clinical characteristics stratified by participating institutionand age group are presented in **Supplementary Table S1**, and corresponding YLL, YLD, and DALY outcomes are presented in **Supplementary Table S2**.

**Table 3:** Multivariable logistic regression analyses of factors associated with premature mortality (YLL > 0).

Variable	Primary model Adjusted OR (95% CI)	p value	Sensitivity model Adjusted OR (95% CI)	p value
Healthcare system: Public	2.15 (1.18–3.94)	0.013	2.00 (1.08–3.73)	0.029
Age at admission, per year	1.01 (1.00–1.03)	0.033	1.01 (1.00–1.03)	0.083
Disease stage: IV	5.74 (3.72–9.09)	<0.001	5.61 (3.63–8.87)	<0.001
BMI, per kg/m²	1.00 (0.97–1.04)	0.814	1.00 (0.97–1.04)	0.861
Ethnicity: Black	3.12 (0.68–22.25)	0.179	3.20 (0.70–22.85)	0.169
Ethnicity: Mixed race	1.49 (0.36–10.15)	0.622	1.51 (0.37–10.28)	0.610
Ethnicity: Uninformed	2.33 (0.60–15.67)	0.287	2.38 (0.61–16.01)	0.277
Ethnicity: White	2.48 (0.68–16.02)	0.235	2.54 (0.70–16.42)	0.222
Smoking status	0.43 (0.23–0.82)	0.011	0.42 (0.22–0.79)	0.009
Alcohol use	0.74 (0.40–1.41)	0.355	0.82 (0.44–1.55)	0.532
Germline testing	—	—	0.56 (0.30–0.97)	0.051
Somatic testing	—	—	4.89 (1.25–15.98)	0.012

Primary model adjusted for healthcare system, age at admission, disease stage, BMI, ethnicity, smoking status, and alcohol use (n = 1940).

Sensitivity model additionally adjusted for germline and somatic testing status (n = 1940).

OR, odds ratio; CI, confidence interval; YLL, years of life lost. Complete-case analysis was used.

**Table 4:** Stratified analysis according to participating institution (public vs private hospital), by age group (<50 and ≥50 years).

Variables		< 50			>= 50			
	N	Private N = 1,184¹	Public N = 166¹	p-value²	N	Private N = 1,515¹	Public N = 237¹	p-value²
Sex	1,350			>0.9	1,752			0.5
Female		1,180 (99.7%)	166 (100.0%)			1,499 (98.9%)	236 (99.6%)	
Male		4 (0.3%)	0 (0.0%)			16 (1.1%)	1 (0.4%)	
Alcohol use	1,350			**<0.001**	1,752			**<0.001**
No		541 (45.7%)	31 (18.7%)			735 (48.5%)	56 (23.6%)	
Yes		643 (54.3%)	135 (81.3%)			780 (51.5%)	181 (76.4%)	
Smoking	1,350			**<0.001**	1,752			**<0.001**
No		504 (42.6%)	32 (19.3%)			634 (41.8%)	45 (19.0%)	
Yes		680 (57.4%)	134 (80.7%)			881 (58.2%)	192 (81.0%)	
Ethnicity	1,350			**<0.001**	1,752			**<0.001**
Asian		1 (0.1%)	16 (9.6%)			8 (0.5%)	20 (8.4%)	
Black		3 (0.3%)	21 (12.7%)			1 (0.1%)	22 (9.3%)	
Mixed race		11 (0.9%)	51 (30.7%)			15 (1.0%)	70 (29.5%)	
Uninformed		967 (81.7%)	8 (4.8%)			1,194 (78.8%)	1 (0.4%)	
White		202 (17.1%)	70 (42.2%)			297 (19.6%)	124 (52.3%)	
Staging	1,350			**<0.001**	1,752			**0.009**
I, II, III		758 (64.0%)	69 (41.6%)			907 (59.9%)	120 (50.6%)	
IV		426 (36.0%)	97 (58.4%)			608 (40.1%)	117 (49.4%)	
BMI	863			**<0.001**	1,077			**<0.001**
Mean (Q1, Q2, Q3)		24.9 (21.6, 24.0, 26.9)	28.9 (24.9, 27.9, 31.8)			26.3 (23.4, 25.7, 28.9)	28.1 (24.3, 27.5, 30.8)	
Min, Max		13.9, 46.0	18.0, 53.1			15.4, 52.7	18.0, 50.2	
Germline Test	1,350			**<0.001**	1,752			**<0.001**
No		908 (76.7%)	155 (93.4%)			1,329 (87.7%)	236 (99.6%)	
Yes		276 (23.3%)	11 (6.6%)			186 (12.3%)	1 (0.4%)	
YLD	1,350	1,099.12	205.32	**<0.001**	1,752	1,451.93	230.35	**<0.001**
YLL	1,350	2,489	717	**0.005**	1,752	2,121.1	593.0	**0.002**
DALY	1,350	3,588	922	**<0.001**	1,752	3,573.0	823.3	**<0.001**

¹n (%); Sum.

²Pearson’s Chi-squared test with simulated p-value (based on 20000 replicates); Wilcoxon rank sum test

BMI, Body Mass Index;

Min, Minimum; Max, Maximum;

Q1, First quartile; Q2, Median; Q3, Third quartile;

YLD, Years Lived with Disability;

YLL, Years of Life Lost;

DALY, Disability-Adjusted Life Years.

Bold values indicate statistically significant results (p < 0.05).

## Discussion

In this retrospective real-world analysis of 3,102 Brazilian patients (2018 to 2023), we evaluated the clinical and demographic profiles of EOBC in patients aged <50 and ≥50 years. The main findings were as follows (1): the proportion of diagnoses varied over time, with a numerical increase of 10% across the study period in EOBC, and a 7.1% reduction in patients aged ≥50 years, with no statistically significant temporal trend (2); there was a similar distribution of clinical stage on diagnosis between both groups (3); younger patients had approximately twice the median YLLs when compared to older patients (median 42 vs 21 years) (4); the aggregate DALY rate per 1,000 patients was 33% higher among younger patients, although patient-level DALY distributions did not differ significantly between age groups.

Recent global analyses have reported an increasing incidence and prevalence of breast cancer among younger patients, despite declining age-standardized mortality and DALY rates (11–13). However, these trends are unevenly distributed. While high Sociodemographic Index (SDI) countries have seen improvements driven by advances in screening and treatment, low- and lower-middle SDI regions, including parts of Latin America, have experienced substantial increases in disease burden – largely due to population growth and limited access to early detection and effective care (13). Cai et al. reported that regions with low and middle SDI showed a significant increase in breast cancer incidence among young women, driven by lifestyle changes, westernized diets, and the adoption of hormonal contraceptives (13). Furthermore, Yuan M identified key contributors to global breast cancer mortality in young women (1990–2029), including alcohol consumption, exposure to secondhand smoke, diets rich in red meat, elevated fasting plasma glucose, physical inactivity, tobacco use, and increased BMI (11). However, these factors were not specifically evaluated as determinants of EOBC or disease burden in the present cohort.

Although the proportion of EOBC diagnoses varied over the study period, no statistically significant temporal trend was identified. The fluctuations observed, particularly the decline in 2020 followed by a rebound in subsequent years, may have been influenced by the COVID-19 pandemic and its effects on healthcare utilization, referral patterns, diagnostic services, and institutional case volume, rather than reflecting true changes in disease occurrence.

Importantly, younger patients had a similar distribution of clinical stage at diagnosis compared with older patients, while the aggregate DALY rate per 1,000 patients was higher in the younger cohort. This difference was primarily driven by the higher aggregate YLL rate. However, patient-level DALY and YLL distributions did not differ significantly between age groups. The higher aggregate YLL and DALY rates reflect the greater number of potential life years lost when death occurs at younger ages and should not be interpreted as evidence of higher mortality risk, more aggressive disease, or poorer survival. Among patients who died during follow-up, the median YLL was twice as high in patients aged <50 years as in those aged ≥50 years (42 vs. 21 years).

Although higher YLL is an inherent consequence of deaths occurring earlier in life, it remains a valuable measure of the societal impact of EOBC because it captures the loss of potential years of life associated with premature mortality. Our findings highlight that the burden of premature mortality in EOBC may be substantial even when the distribution of clinical stage at diagnosis is similar across age groups. These findings support continued attention to age-appropriate breast cancer care and guideline-based genetic counseling and testing, particularly given the higher proportion of germline testing observed among younger patients in our cohort.

We also observed differences between the two participating institutions with respect to the probability of YLL >0. After adjustment for age at admission, clinical stage, BMI, ethnicity, smoking status, and alcohol use, treatment at the public institution remained associated with higher odds of YLL >0. However, these findings should be interpreted as adjusted institutional associations rather than effects of the healthcare sector itself, as each sector was represented by a single participating institution.

This study has several limitations. First, comparisons between the participating institutions should not be interpreted as causal effects of the healthcare sector, as each healthcare sector was represented by a single institution. Institutional differences in referral patterns, patient case mix, treatment availability, follow-up, and data completeness may have influenced the observed findings.

Furthermore, although the regression analysis evaluating YLL >0 included adjustment for several demographic and clinical variables, residual confounding cannot be excluded because of the observational design and the lack of information on important prognostic factors. Calendar year of diagnosis and follow-up duration were not included as covariates in the multivariable model evaluating YLL >0. This is particularly relevant because follow-up duration varied across the study period, with patients diagnosed in later years having less time available for observation. Consequently, these patients had less opportunity for death ascertainment during the study period, which may have influenced mortality-related findings and resulted in lower observed YLL estimates, particularly among patients with earlier-stage disease. Therefore, residual confounding related to calendar time and differential follow-up cannot be excluded, and the mortality and YLL findings should be interpreted with caution.

Second, the study was based on data from only two institutions in the state of São Paulo, Brazil, which may limit the generalizability of the findings to other Brazilian regions, where significant disparities in disease burden and risk factors have been documented (14).

Third, although DALYs and YLLs were calculated using individualized life expectancy projections, YLD estimates relied on simplified stage-specific disability weights derived from the Global Burden of Disease methodology and did not account for transitions between treatment phases, remission, recurrence, or disease progression. Furthermore, because YLD was calculated using the observed duration of follow-up, patients diagnosed earlier accumulated longer disability time than those diagnosed more recently. These methodological assumptions may have influenced individual YLD estimates. Additionally, individual information for stages I-III was not available in the extracted dataset. Consequently, disease stage was analyzed as non-metastatic (stages I-III) versus metastatic (stage IV), limiting more detailed stage-specific analyses. In addition, BMI, ethnicity, staging, and germline testing data may have been influenced by differences in documentation practices between the participating institutions. In particular, BMI data were unavailable for a substantial proportion of patients, and ethnicity information was frequently unreported, especially in one participating institution. Consequently, analyses involving these variables may be subject to information bias and should be interpreted with caution.

Fourth, histological and molecular subtypes were not assessed. Despite their known prognostic significance, triple-negative and HER2-positive subtypes are more prevalent in younger patients and associated with worse outcomes, while the recent rise in EOBC is thought to be largely driven by increased diagnosis of luminal tumors (15, 16). Additionally, genetic cases were not excluded, and the cohort was not stratified by treatment modality (e.g., anthracycline-based chemotherapy, radiotherapy), limiting evaluation of the interaction with treatment exposures (17). Furthermore, germline testing results, pathogenic variant frequencies, indications for testing, and potential differences in access to testing between the participating institutions were not evaluated. Therefore, no conclusions can be drawn regarding mutation prevalence or unmet needs for genetic services. Finally, potentially relevant risk factors, such as hormone replacement therapy, reproductive factors, and other pharmaceutical exposures, were not available in the dataset and could not be evaluated. Their absence may have contributed to residual confounding.

Further research is needed to better understand the biological, clinical, and systemic factors associated with the burden of EOBC observed in this institutional cohort. Although younger patients had a similar distribution of clinical stage at diagnosis, they had higher aggregate YLL and DALY rates, primarily driven by YLL. These findings reflect the greater number of potential life years lost when death occurs at younger ages and should not be interpreted as evidence of higher mortality risk or more aggressive disease. Prospective multicenter studies with longer and standardized follow-up, detailed tumor subtype and treatment information, and more comprehensive assessment of prognostic and social factors are needed to further characterize the determinants of outcomes and disease burden in younger patients.

## Conclusion

Our findings demonstrate variation in the proportion of EOBC diagnoses within two tertiary cancer centers in São Paulo, Brazil, and show that younger patients had higher aggregate YLL and DALY rates, reflecting the greater number of potential life years lost when deaths occur at younger ages, despite a similar distribution of non-metastatic (stages I-III) versus metastatic (stage IV) disease at diagnosis. The relatively high proportion of stage IV disease likely reflects the referral patterns of the participating tertiary cancer centers, which receive a substantial number of patients referred after diagnosis from other institutions. Therefore, the stage distribution observed in this cohort should not be interpreted as representative of the general Brazilian breast cancer population. These findings support further research into age-specific clinical care and the biological and social determinants of breast cancer in younger patients.

## Data Availability

The raw data supporting the conclusions of this article will be made available by the authors, without undue reservation.

## Supplementary material

Supplementary Table 1 Demographic and clinical characteristics stratified by participating institution and age group. Data are presented as number and percentage for categorical variables and as median (Q1–Q3) and minimum–maximum for body mass index (BMI). The “Available N/Missing” column indicates the number of patients with available data and the number and percentage of missing observations for each variable. Comparisons between the participating private and public institutions were performed separately within each age group (<50 and ≥50 years) using Pearson’s chi-squared test with simulated p-values based on 20,000 replicates for categorical variables and the Wilcoxon rank-sum test for continuous variables. BMI , body mass index; Q1 , first quartile; Q3 , third quartile.

Supplementary Table 2 Disease burden outcomes stratified by participating institution and age group. For each outcome, the first row presents the patient-level median (Q1-Q3), and the second row presents the aggregate total for the participating private and public institutions within each age group (<50 and ≥50 years). Comparisons of patient-level outcome distributions between the participating institutions were performed separately within each age group using the Wilcoxon rank-sum test. The aggregate totals are descriptive and were not included in the statistical comparisons. YLD, Years Lived with Disability; YLL, Years of Life Lost; DALY, Disability-Adjusted Life Years; Q1, first quartile; Q3, third quartile.
